# Protein Neighbors and Proximity Proteomics*[Fn FN2]

**DOI:** 10.1074/mcp.R115.052902

**Published:** 2015-09-08

**Authors:** Johanna S. Rees, Xue-Wen Li, Sarah Perrett, Kathryn S. Lilley, Antony P. Jackson

**Affiliations:** From the ‡Department of Biochemistry, University of Cambridge, Tennis Court Road, Cambridge, United Kingdom CB2 1QW,; the §Cambridge Centre for Proteomics, University of Cambridge, Tennis Court Road, Cambridge, United Kingdom CB2 1QR, and; the ‖National Laboratory of Biomacromolecules, Institute of Biophysics, Chinese Academy of Sciences, 15 Datun Road, Beijing 100101, China

## Abstract

Within cells, proteins can co-assemble into functionally integrated and spatially restricted multicomponent complexes. Often, the affinities between individual proteins are relatively weak, and proteins within such clusters may interact only indirectly with many of their other protein neighbors. This makes proteomic characterization difficult using methods such as immunoprecipitation or cross-linking. Recently, several groups have described the use of enzyme-catalyzed proximity labeling reagents that covalently tag the neighbors of a targeted protein with a small molecule such as fluorescein or biotin. The modified proteins can then be isolated by standard pulldown methods and identified by mass spectrometry. Here we will describe the techniques as well as their similarities and differences. We discuss their applications both to study protein assemblies and to provide a new way for characterizing organelle proteomes. We stress the importance of proteomic quantitation and independent target validation in such experiments. Furthermore, we suggest that there are biophysical and cell-biological principles that dictate the appropriateness of enzyme-catalyzed proximity labeling methods to address particular biological questions of interest.

## INTRODUCTION: THE CROWDED CELL

**Fig. 1.**
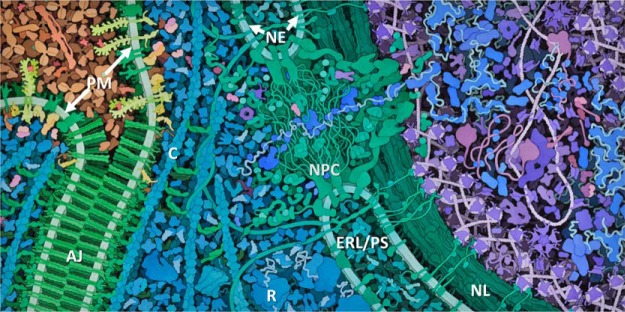
**An example to illustrate the crowded nature of the cell.** Here, two adjacent vascular endothelial cells are shown connected via an adherens junction (*AJ*) between the opposing plasma membranes (*PM*) of the two cells. The adherens junction contains localized clusters of cell adhesion proteins, mainly cadherins and catenins (*green*). Note that other plasma membrane proteins such as vascular endothelial growth factor receptors (*yellow*) are excluded from the clusters. Extracellular blood plasma proteins are shown in *tan* (*upper left*). Cytoplasmic proteins are shown in *turquoise*, and nuclear proteins are shown in *purple* and *blue* (*right*). The image also displays common features of the eukaryotic cell, including ribosomes (*R*), cytoskeletal cables (*C*), the nuclear pore complex (*NPC*), the nuclear lamina (*NL*), the nuclear envelope (*NE*), and the ER lumen/perinuclear space (*ERL/PS*). The macromolecules are shown at their approximate true densities and molecular dimensions. For more details about the biological context of this image, see *Span* et al. (73) (used with permission of David S. Goodsell, the Scripps Research Institute).

Cellular proteins typically exist within a highly crowded environment (Fig. 1). This striking feature has important implications for many aspects of molecular cell biology, including protein folding, protein mobility, enzyme kinetics, and gene expression (1–3). In particular, macromolecular crowding has probably facilitated the evolution of extended weak but functionally important protein-protein interactions (4). For example, studies of the protein interactome of brewers' yeast *Saccharomyces cerevisiae* imply the existence of many core or “hub” protein complexes, which also transiently bind to a larger range of proteins, many of which are shared between different hubs (5). This dynamic but structured behavior has been called the “molecular sociology of the cell” (6). The effect is particularly evident on membrane surfaces where the reduction from three to two spatial dimensions significantly decreases the binding affinities required to maintain stable protein-protein interactions. Here, contacts between individual membrane proteins can build up to produce more extended protein clusters of restricted composition and indeterminate stoichiometry (7). Examples include the assembly of ion channels on the neuronal plasma membrane (8) and the molecular components of cell junctions (Fig. 1). Similar examples for intracellular proteins include the binding of multiple transcription factors to localized regions of DNA (9) and the assembly of multiple signaling proteins onto the cytoskeleton (10).

It is a characteristic feature of all these cases that any one protein will typically interact only with its immediate neighbors but usually not with all the proteins in the cluster. However, it is the overall *proximity* of the proteins within the network, not just the immediate binding partners of the proteins, that is likely to be functionally important. Many individual interactions that are significant *in vivo* have dissociation constants in the tens to hundreds of micromolar range and fast off-rate constants (11). Following the dramatic reduction in protein concentration after detergent-mediated cell lysis, these interactions will be lost too quickly to be detected by methods such as immunoprecipitation or tandem affinity purification tagging. Chemical cross-linking has been extensively used to probe protein-protein interactions (12, 13). However, this can be difficult to control with too much cross-linking producing large insoluble complexes (14). More recent developments have exploited reagents that can be selectively targeted to a protein of interest and then photoactivated to tag the binding partners of the protein (15). However, cross-linking and photoactivatable reagents will typically span a distance of only about 4–15 Å (16). Therefore all of these approaches are best suited to the analysis of relatively simple complexes. The challenge is to develop additional proteomic methods that can identify and systematically characterize proteins within larger spatially restricted but weakly interacting multicomponent complexes as they occur *in vivo*.

## ENZYME-CATALYZED PROXIMITY PROTEOMICS: CHEMISTRY AND APPLICATIONS

**Fig. 2.**
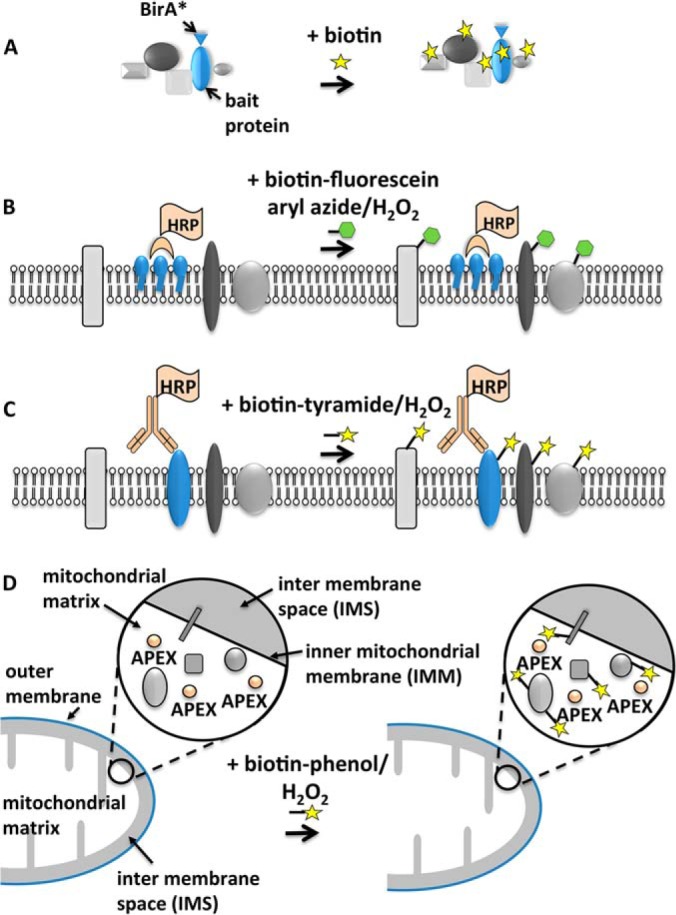
**Summarized outline of the major published enzyme-catalyzed proximity labeling assays.** *A*, in BioID (20), a bait protein fused to a promiscuous biotin protein ligase (*blue*) is expressed in cells. Biotin is added to initiate biotinylation of closely associated proteins (*gray*). *B*, EMARS (17). In this example, HRP-coupled cholera toxin (*tan*) is added to cells and binds lipid raft-associated gangliosides in the plasma membrane (*blue*). Fluorescein- or biotin-conjugated aryl azide with hydrogen peroxide initiates labeling of neighboring proteins (*gray*). *C*, SPPLAT (19). HRP-coupled antibody (*tan*) to a plasma membrane target protein (*blue*) is added to cells. Biotin-tyramide with hydrogen peroxide initiates labeling of neighboring proteins (*gray*). *D*, use of APEX for organelle-specific labeling. Here, APEX (*tan*) has been engineered to be selectively expressed in the mitochondrial matrix as described (18). Cells are briefly incubated with a biotin-tyramide derivative (biotin-phenol) with hydrogen peroxide to initiate labeling of matrix-associated proteins (*gray*). In all cases, cells are lysed, and labeled proteins are isolated by affinity pulldown using immobilized streptavidin (for biotinylated proteins) or anti-fluorescein antibody (for fluorescein-tagged proteins). Samples are analyzed by mass spectrometry.

In the last few years, several groups have independently explored the potential for enzyme-catalyzed “proximity labeling” as a general tool for the proteomic characterization of extended protein clusters and weakly or transiently associating protein complexes and as a means of characterizing proteins within spatially restricted intracellular compartments (14, 17–21). The basic concept is remarkably simple and exploits the behavior of some enzymes to generate a small, unstable reagent that is capable of covalently labeling a protein target. The short half-life of the enzyme-generated product ensures that only proteins in the immediate vicinity of the enzyme (typically a few tens to hundreds of nanometers; see below) are covalently modified (22–24). If the enzyme can be directed to a specific protein or cellular compartment of interest and if the labeling reagent contains a molecular tag such as biotin that enables easy purification, then in principle the immediate neighbors of the protein can be marked for later isolation by standard pulldown methods and then analyzed by mass spectrometry (Fig. 2). This approach has the added advantage that the labeling can be performed in living cells, allowing physiologically relevant interactions to be investigated even when they are weak or transient. The following methods represent different applications of this core idea.

## PROXIMITY LABELING USING BIOTIN PROTEIN LIGASE

The *Escherichia coli* enzyme BirA is a biotin protein ligase that normally catalyzes the endogenous biotinylation of a specific lysine residue on acetyl-CoA carboxylase (25). However, BirA will also biotinylate a lysine residue when it occurs within the context of other short “acceptor peptide” sequences (26). Specific protein-protein interactions can then be investigated if the BirA is fused to one protein and co-expressed together with a second protein containing an engineered acceptor peptide sequence. Here, biotinylation of the acceptor peptide-tagged protein will occur if the two proteins are in close association (27). This approach can be readily adapted for use with mass spectrometry (21, 28). Assays of this type are most suitable for use with hypothesis-driven experiments where interacting partners are already known or strongly suspected (14). To apply BirA in a more discovery-based context, the enzyme must be modified. BirA combines biotin with ATP to produce biotinoyl-5′-AMP. This reactive and unstable intermediate is normally held at the active site until it is transferred to its target protein (29). However, a mutant biotin protein ligase called BirA* nonspecifically biotinylates any protein with exposed lysine residues that lies in the immediate vicinity of the enzyme. Although the precise mechanism is not yet known, the most likely reason for this altered behavior is that the BirA* mutant prematurely releases biotinoyl-5′-AMP into the medium (14, 30). Roux *et al.* (20, 31) exploited BirA* to develop *proximity-dependent biotin identification* (BioID)^1^ (Fig. 2*A*). In the first application of this method, BirA* was fused to nuclear lamin A, a major cytoskeletal component of the nuclear lamina (Fig. 1). When the chimera was expressed in HEK293 cells supplemented with exogenously added biotin, it catalyzed the *in vivo* biotinylation of about 100 proteins, which were then isolated and identified by mass spectrometry. The majority of the biotinylated proteins were nucleus-associated. They included several known nuclear lamina-binding proteins together with previously uncharacterized proteins. The most abundant of the unknown proteins were localized to the nuclear envelope and are strong candidates for novel lamin interactors (20). BioID has been used successfully in a growing number of examples, including centrosome components (32), the nuclear pore complex (33), c-Myc-interacting partners in tumor cells (34), the inner membrane complex of *Toxoplasma gondii* (35), chromatin-associated proteins (36), tight junction proteins in Madin-Darby canine kidney epithelial cells (37) and the bilobe, a poorly characterized cytoskeletal component in *Trypanosoma brucei* (38).

## PROXIMITY LABELING USING PEROXIDASE ENZYMES

In the presence of peroxidase enzymes, phenolic compounds such as tyramine or phenolic aryl azide derivatives react with hydrogen peroxide to generate a short lived free radical (39, 40). For tyramine, the enzyme-generated reagent can covalently label exposed aromatic groups on proteins such as the side chains of tyrosine and tryptophan residues, although side chains of other amino acids such as histidine and cysteine may also be labeled (22, 41). Biotin and fluorescent amide derivatives of tyramine or aryl azide can easily be synthesized (17, 42). This has led to the widespread use of enzyme-catalyzed proximity labeling as an amplification method in immunohistochemistry, immunoassays, and *in situ* hybridization (43–45). The first application of the method to proteomics exploited the presence of endogenous membrane-bound peroxidases in neutrophils (46) and fertilized eggs of the sea urchin *Strongylocentrotus purpuratus* (47). In both cases, fluorescently labeled tyramide derivatives were used to identify neighboring proteins of these endogenous enzymes. However, to apply the method more generally requires that the peroxidase be intentionally directed to a particular protein or cellular compartment of choice.

### 

#### 

##### Enzyme-mediated Activation of Radical Sources (EMARS)

In this method, horseradish peroxidase (HRP) is coupled to an antibody or a protein ligand that binds a plasma membrane molecule (17, 48, 49) (Fig. 2*B*). Alternatively, the HRP can be expressed as a fusion protein with a targeting signal that directs the enzyme to a plasma membrane subdomain (50). The labeling reagent is either aryl azide-biotin or aryl azide-fluorescein. The EMARS method has been particularly helpful in the proteomic analysis of lipid rafts. These plasma membrane-based structures are attractive candidates for study using enzyme-catalyzed proximity labeling. They have a distinct but poorly annotated composition, they have dimensions comparable with the footprint of the peroxidase-generated labeling reagent, and they play important roles in cell signaling and membrane sorting (51). HRP-coupled cholera toxin was used to bind the raft ganglioside GM1. Proteomic analysis identified proteins known to co-cluster with GM1 such as CD44 and integrins together with other proteins known to be implicated in signal transduction pathways. About 10% of the detected proteins were cytosolic peripheral proteins, suggesting that the enzyme-generated aryl azide free radical may cross the membrane (17). This is not necessarily a problem provided the diffusion distance through the membrane is limited to the immediate membrane undersurface. Indeed, it may be an advantage because distinct cytoskeletal and peripheral proteins can associate with lipid rafts and other plasma membrane protein clusters (see below).

##### Selective Proteomic Proximity Labeling Assay using Tyramide (SPPLAT)

SPPLAT is a proximity labeling method in which an HRP-coupled antibody or protein ligand to a plasma membrane protein is added exogenously to cells (19, 52) (Fig. 2*C*). The proximity labeling reagent contains a tyramide moiety connected to biotin via a 12-carbon atom spacer arm containing a disulfide bond. The arm ensures that the biotin is accessible to the streptavidin matrix used for purification, and the disulfide bond facilitates easy recovery from the affinity matrix by elution with reducing agent (19). SPPLAT has been used to examine the proteins that co-assemble with the activated B-cell receptor (BCR) on the plasma membrane of the B-lymphocyte cell line DT40. BCRs on the surface of B lymphocytes can be cross-linked by bivalent anti-BCR antibodies. This behavior is known to mimic antigen-induced BCR cross-linking, and in DT40, it promotes the co-assembly of BCR molecules with other molecules implicated in BCR signaling (53). An HRP-coupled anti-BCR antibody can therefore both drive the clustering of the BCRs on the plasma membrane and enable the co-assembled molecules to be biotinylated using the biotin-tyramide reagent. The cross-linked BCR molecules assemble into asymmetrically distributed clusters on the B-cell plasma membrane (53, 54). This enables the close correlation between the BCR and deposited biotin to be readily confirmed by immunolocalization in both two (19) and three dimensions (supplemental Figs. S1A–S1C). A SILAC-based quantitative proteomic analysis identified known neighbors of the cross-linked BCR but also revealed new aspects of the process, including the co-clustering of immunoglobulin family proteins previously of unknown function but now linked with BCR-activated integrin signaling (19). Most of the identified proteins were intrinsic membrane components. However, a small number were proteins known to associate peripherally with the cytosolic face of the plasma membrane. Indeed, one cytoplasmic membrane-associated protein, cdc42, showed very weak but detectable biotinylation (19). This indicates that, as with the EMARS reagent, the enzyme-generated tyramide radical may be able to cross the plasma membrane at least to a limited extent. Interestingly, one of the identified peripheral proteins, guanine nucleotide-binding protein G_i_ subunit α2 was also detected in the EMARS analysis of lipid rafts (17). Because the cross-linked BCR is known to enter lipid rafts (55), this supports the view that guanine nucleotide-binding protein G_i_ subunit α2 may be a novel marker for these membrane structures.

##### Proximity Labeling with Ascorbate Peroxidase (APEX): a Method to Identify Proteins within an Organelle

For eukaryotic cells in particular, distinct proteins are often sequestered into different spatially restricted, membrane-bound intracellular compartments (Fig. 1). Proximity labeling thus offers a novel approach for the proteomic characterization of organelles or even organelle subcompartments. It should be noted that in this particular application, unlike the previous examples, the primary aim is not necessarily to provide detailed maps of closely interacting protein complexes (although this could certainly be achieved). Rather, it is to selectively label as many of the compartment-specific proteins as possible. Hence, the proximity labeling enzyme must be targeted to the organelle of interest. It should be expressed without attachment to other proteins so that it is distributed evenly throughout the compartment and free to diffuse within the membrane-bounded organelle. It should also be expressed at a level necessary to ensure efficient protein labeling. Rhee *et al.* (18) used a monomeric APEX for this purpose. When expressed with a mitochondrial targeting signal in HEK293T cells, the APEX enzyme was selectively inserted into the mitochondrial matrix (Fig. 2*D*). To initiate labeling, cells were incubated with hydrogen peroxide and a biotinylated tyramide derivative (biotin-phenol), both of which are membrane-permeant. Using SILAC-based quantitative proteomics, 495 proteins were identified within the human mitochondrial matrix, including 31 not previously linked to mitochondria. The labeling was exceptionally specific and could distinguish between proteins localized to the inner mitochondrial membrane that faced the matrix *versus* those inner mitochondrial membrane proteins facing the intermembrane space (IMS) (Fig. 2*D*). On the basis of these experiments, several proteins previously misassigned to the IMS were reassigned to the matrix, and their location was confirmed by electron microscopy (18). Of course, rather than simply expressing free APEX within an intracellular compartment, the enzyme could also be fused to a specific protein of interest (18, 56). This would enable mapping experiments to be carried out that target particular intracellular protein complexes or organelle subdomains.

## ENZYME-CATALYZED PROXIMITY PROTEOMICS: CONSTRAINTS AND CONTROLS

Enzyme-catalyzed proximity labeling differs conceptually from traditional approaches such as immunoprecipitation or cross-linking because the labeled proteins may not interact with the enzyme-targeted protein directly but merely lie within a limited distance from the target. For applications that aim to define specific protein complexes, this is both a strength and a potential problem for the technique. Here, a critical question is the size of the labeling footprint in comparison with the likely size of the molecular complex being investigated: a footprint that is too small risks many false negatives, whereas one that is too big risks many false positives. The footprint radius in a typical experiment will depend on a number of factors, including the half-life of the enzyme-generated labeling reagent and the incubation time. Using quantitative immunoelectron microscopy, the footprint radius for tyramide-based reagents under realistic labeling conditions has been measured at up to 40 nm (18, 57). Similar experiments suggest a footprint radius of up to 200 nm for aryl azide-based labels (24). To investigate the footprint radius of BioID, Kim *et al.* (33) examined the nuclear pore complex. This is a large and stable structure localized to the nuclear envelope and whose subunit dispositions and approximate overall dimensions are known (Fig. 1). By expressing different BirA*-subunit fusion proteins and detecting biotinylation of the associated subunits, the nuclear pore complex was used as a convenient “molecular ruler.” They reported an effective footprint radius of about 10 nm (33). However, it should be noted that BioID labeling acts through a biotin-adenylate ester, which has a longer half-life than tyramide-based reagents (18), and the method also requires relatively long incubation times (20). On this basis, the footprint radius estimated by Kim *et al.* (33) is surprisingly small.

For an enzyme-tagged protein complex that is tethered within the cell, the footprint volume is proportional to the cube of the footprint radius. However, in the crowded intracellular environment (Fig. 1), the mean distance between proteins has been calculated to be less than 10 nm (58). So even a footprint radius in the tens of nanometers range will risk generating nonspecifically biotinylated proteins. Moreover, an enzyme-tagged protein complex that is not tethered will be free to diffuse within the cell. Even with large multisubunit assemblies, this diffusion can be significant over the time scales of a typical labeling experiment. For example, the diffusion constant for the mobile fraction of the large ribosomal subunit in rat myoblasts has been measured at 0.31 μm^2^ s^−1^ (59). Hence, in 1 min (the shortest reported labeling time), an enzyme-tagged protein complex of this size will likely diffuse about 10 μm, or about half the width of a typical cell, all the while spraying enzyme-generated labeling reagent along its path. It can be argued that the problem will be less severe with proteins anchored into extended complexes on membrane or cytoskeletal surfaces because they typically have lower diffusion constants (60). Yet even here, an enzyme bound at the edge of a cluster will likely “bleed” labeling reagent onto proteins not associated with the cluster. Crucially, however, proteins that remain in close proximity to an enzyme-tagged neighbor during the experiment will be more strongly labeled than other proteins that only interact randomly and fleetingly due to molecular crowding. For this reason, proteomic quantitation should be applied in conjunction with enzyme-catalyzed proximity labeling experiments to help identify true neighbors.

Quantitative experiments have so far used a SILAC-based approach (18, 19, 56), although spectral counting has been used as a semiquantitative method in some BioID assays (32, 33, 35). Importantly, quantitation can discern different degrees of association between proteins. For example, in the SPPLAT analysis of the BCR clusters on DT40 lymphocytes, cells grown in heavy SILAC medium were incubated with HRP-coupled anti-BCR antibody, and cells incubated in light SILAC medium were incubated with an HRP-coupled nonspecific antibody of the same Ig class and isotype. The experiment was then repeated with a reciprocal incubation protocol. Proteins with the most significant isotope ratios (reflecting selective enrichment for BCR-associated proteins) were all plasma membrane-localized. However, there was also a clear separation of SILAC ratios for different classes of organelle proteins. As expected, nuclear proteins showed the lowest SILAC ratios for specifically biotinylated *versus* nonspecifically biotinylated proteins. Interestingly, however, mitochondrial and some cytoskeletal proteins had somewhat higher ratios, suggesting that these structures were closer to the BCR cluster. Indeed, mitochondria did accumulate under the BCR clusters in these cells (19).

The power of SILAC quantitation in a proximity labeling experiment has been strikingly demonstrated in a recent report by Hung *et al.* (56). These workers focused on the mitochondrial IMS, a subcompartment whose proteomic composition has been difficult to characterize. Here, they used an IMS-targeted APEX enzyme to biotinylate the IMS proteins. The IMS is bounded by the inner and outer mitochondrial membranes (Fig. 2*D*). Unfortunately, the mitochondrial outer membrane contains porins that make it freely permeable to molecules below about 5 kDa in molecular mass. Thus the APEX-generated free radical will inevitably diffuse out of the mitochondria. Because the outer mitochondrial membrane is only about 5 nm thick, some labeling of cytosolic proteins is unavoidable (56). To resolve this problem, Hung *et al.* (56) used an ingenious experimental design. HEK293T cells expressing an IMS-targeted APEX were grown in heavy (H) isotope culture; cells expressing a cytosol-targeted APEX were grown in medium (M) isotope culture, and control cells without APEX were grown in light (L) isotope culture. Proteins with a high H/L ratio are predominantly biotinylated by the IMS APEX; those with a high M/L ratio are predominantly biotinylated by the cytosolic APEX. The H/M ratio for a given protein will reflect the relative extent to which it is biotinylated by the IMS APEX *versus* the cytosolic APEX. For example, a true IMS protein should show a high H/L ratio, a low M/L ratio, and a high H/M ratio. Conversely, a cytosolic protein that is artifactually biotinylated by IMS APEX should show a high H/L ratio, a high M/L ratio, and a low H/M ratio. It should be noted that the H/M ratio will reflect only the relative proximity of a particular protein to the IMS or the cytosol because for a given protein all other factors (such as the steric accessibility of individual protein tyrosine residues) will affect IMS and cytosolic labeling to the same extent. The method clearly identified a population of molecules consistent with true IMS-located proteins and could effectively discriminate them from cytosolic proteins (56).

A further issue is the nature of the labeling enzyme. It must be sufficiently active to generate enough labeled protein to be isolated, but the added bulk of the enzyme must not unduly compromise its incorporation into the larger protein complex. For cases such as SPPLAT and EMARS where the labeling enzyme is directed to the extracellular face of cell surface protein clusters, this latter concern is probably less critical (17, 19, 52). Here, HRP is the enzyme of choice as it has excellent activity and stability profiles for radical-based labeling reagents. Unfortunately, HRP misfolds when expressed in many intracellular compartments. Hence, APEX was developed as an alternative intracellular labeling enzyme (61). However, the original APEX enzyme used by Rhee *et al.* (18) suffered from a number of drawbacks. In particular, its activity was comparatively poor, and the enzyme was oxidatively damaged by hydrogen peroxide at the concentration used in labeling experiments. As a result, the enzyme needed to be expressed at relatively high concentrations within organelles, which in some cases led to protein aggregation (18). To circumvent this problem, Lam *et al.* (62) used directed evolution to produce a modified enzyme (APEX2) that is more active and less sensitive to oxidative damage than the original enzyme and is thus better suited to *in vivo* proximity labeling. The chimeric addition of the enzyme to a protein might sometimes interfere with folding. In a proximity labeling experiment, this would lead to a false negative result or even spurious biotinylation if the enzyme-tagged protein was mistargeted. The APEX enzyme is comparable in size to green fluorescent protein, whereas BirA* is somewhat larger (14). Some proteins tagged with green fluorescent protein do indeed misfold (63), although this occurs in a relatively small number of cases (64). Thus, protein misfolding of chimeric molecules is a real albeit relatively rare possibility. We therefore stress the importance of controls to establish correct folding and targeting of the chimeric protein on a case-by-case basis.

A final consideration is that of target accessibility. Free radical tyramide-based reagents covalently couple to only a small number of amino acid side chains, mainly aromatic groups such as tyrosine (41). Labeling is therefore likely to be relatively infrequent, and it will critically depend on side-chain exposure, which may be restricted due to macromolecular packing within protein assemblies. Furthermore, it appears that for tyramide-based reagents, it is not so easy to detect the individually modified peptides. The reasons are not well understood, but it has been suggested that the underlying chemistry of tyramide labeling may generate many more heterogeneous adducts than initially anticipated (52, 56). Here, BioID may have an advantage because the method labels lysine residues with a better defined chemistry (65). In addition, lysines are more abundant and tend to be more solvent-exposed than aromatic amino acids. If labeled peptides can be identified, they should provide valuable insights into not only the structural disposition of proteins within membrane-bound complexes but also their degree of exposure to the intracellular medium. For example, Rhee *et al.* (18) detected specific peptides containing biotinylated tyrosines from three mitochondrial matrix and inner mitochondrial membrane proteins (pyruvate dehydrogenase, 3,2-*trans*-enoyl-CoA isomerase, and acetoacetyl-CoA thiolase). Because the atomic resolution structures of all three proteins are known, the location of the modified tyrosine residues could be identified on the surfaces of each molecule. As expected, the modified tyrosines were all exposed on domains of the proteins that face toward the inner matrix space (18).

In light of the limitations noted above, it is particularly important to provide independent verification for candidate proteins identified by proximity labeling. Immunofluorescence co-localization, although necessary, is not on its own sufficient. Rather, confirmatory experiments should directly address the issue of protein proximity because this is the aspect that is explicitly being investigated in these proteomics experiments. Because APEX was originally developed as an enzyme for use in high resolution electron microscopy (61), it can be used both to mark proteins in live cells for proteomic analysis and for subsequent electron microscopy validation studies. Another possibility is optical super-resolution imaging (66), but this requires specialized equipment. An easier approach is the proximity ligation assay (67). This immunological method is simple to perform and can identify proteins that lie up to about 40 nm from each other (19), which is comparable with the size range likely to be detected by enzyme-catalyzed proximity labeling methods. In some cases, there will be prior information on the types of proteins expected within a protein assembly, and this can provide additional help for the interpretation of proteomic results. However, it should be borne in mind that existing data may have been provided by experiments that do not detect indirectly associating proteins. For example, in an interesting experiment, immunoprecipitation and BioID were directly compared for the interactome maps of chromatin-associated protein complexes. As expected, BioID produced a larger data set of potentially interacting molecules and tended to detect partners of lower cellular abundance, but only in a few cases were the same partners detected by both methods (36).

## CONCLUSIONS AND FUTURE PROSPECTS

Enzyme-catalyzed proximity labeling is a new approach to proteomics, and there are a number of areas where methodological advancement would be welcome. For example, alternative methods of proteomic quantitation are probably required for those cases such as slow growing cells or tissue and organ slices where SILAC is impractical (68). Additional labeling chemistries to target a wider range of amino acid side chains would further improve protein coverage, and other labeling enzymes such as lipoic acid ligase (69) should further broaden the experimental options.

The use of enzyme-catalyzed proximity labeling to characterize mitochondrial compartment proteomes (18, 56) could certainly be extended to other organelles. However, one complication is that many organelles, particularly within the secretory pathway, exchange component proteins. For example, the luminal steady-state “resident” proteins of the endoplasmic reticulum (ER) (Fig. 1) constantly leave the organelle but then quickly return from post-ER compartments because they contain a carboxyl-terminal retrieval sequence (70). If enzyme-catalyzed proximity labeling were used to characterize the ER-resident proteome, then an ER-targeted APEX enzyme would be transiently exposed to post-ER compartments. Hence, some form of triple isotope SILAC experiment (see above) using cells separately expressing ER-targeted and post ER-targeted APEX would probably be needed to identify true resident ER proteins.

As well as studying the steady-state composition of organelles, enzyme-catalyzed proximity labeling might also be useful to examine dynamic aspects of protein behavior. For example, a labeling reaction catalyzed by an enzyme-coupled protein *in vivo* will leave a close neighboring protein with an attached covalent tag. This tag will persist even if the two proteins subsequently move apart. Thus, it may be possible to sequentially tag the different neighbors of a protein as it moves through consecutive membrane trafficking compartments within the secretory or endocytic pathways. In provisional experiments, we have shown that a pulse of HRP-linked transferrin applied with membrane-permeant biotin-tyramide and hydrogen peroxide can differentially label membrane proteins of the endosomal pathway in a temporal sequence (52). In such experiments, the results could then be compared with data from other methods that record whole organelle proteomes such as localization of organelle proteomes by isotope tagging (71, 72). This would place the itinerary-specific proteome within its broader cell-biological context.

In summary, the use of enzyme-catalyzed proximity proteomics has already moved beyond the “proof of concept” stage. It is now beginning to provide significant new insights into a variety of cell-biological questions, including protein-protein assembly, cell signaling from membrane-bound receptors, and organelle proteomics. However, the full potential for these types of assays is still to be realized.

## Supplementary Material

Supplemental Data

## Footnotes

^1^ The abbreviations used are:

BioID: proximity-dependent biotin identification
APEX: ascorbate peroxidase
BCR: B-cell receptor
EMARS: enzyme-mediated activation of radical sources
ER: endoplasmic reticulum
HRP: horseradish peroxidase
IMS: intermembrane space
SILAC: stable isotope labeling of amino acids in culture
SPPLAT: selective proteomic proximity labeling assay using tyramide
GM1: monosialotetrahexosylganglioside
H: heavy
L: light
M: medium.
