# Supplementary material for: Protein Neighbors and Proximity Proteomics
Source: Mol Cell Proteomics. 2015 Sep 8;14(11):2848–56. doi: 10.1074/mcp.R115.052902 (PMC4638030; doi:10.1074/mcp.R115.052902)
Supplement: Supplemental Data [file supp_14_11_2848__index.html]

Protein Neighbors and Proximity Proteomics — Protein Neighbors and Proximity Proteomics — Protein Neighbors and Proximity Proteomics — Supplemental Data 

# Protein Neighbors and Proximity Proteomics

## Supplemental Data

- Supplementary Figure S1 - Single frames of 3D rendered confocal images (ImarisBitplane) for DT40 B-lymphocyte cells stained for: A) B-cell receptor, B) SPPLAT-deposited biotin, following incubation with HRP-tagged anti-(B-cell receptor) antibody, C) Merged images of A) and B).
- Supplementary Figure S1A - 3D rendered confocal images (ImarisBitplane) for DT40 B-lymphocyte cells stained for the B-cell receptor.
- Supplementary Figure S1B - 3D rendered confocal images (ImarisBitplane) for DT40 B-lymphocyte cells stained for SPPLAT-deposited biotin, following incubation with HRP-tagged anti-(B-cell receptor) antibody.
- Supplementary Figure S1C - 3D rendered confocal images (ImarisBitplane) for DT40 B-lymphocyte cells: Merged images of Figure S1A and S1B.
