# Supplementary material for: Protein Neighbors and Proximity Proteomics
Source: Mol Cell Proteomics. 2015 Sep 8;14(11):2848–56. doi: 10.1074/mcp.R115.052902 (PMC4638030; doi:10.1074/mcp.R115.052902)
Supplement: Supplemental Data [file supp_R115.052902_mcp.R115.052902-1.pdf]

Supplementary Fig. S1

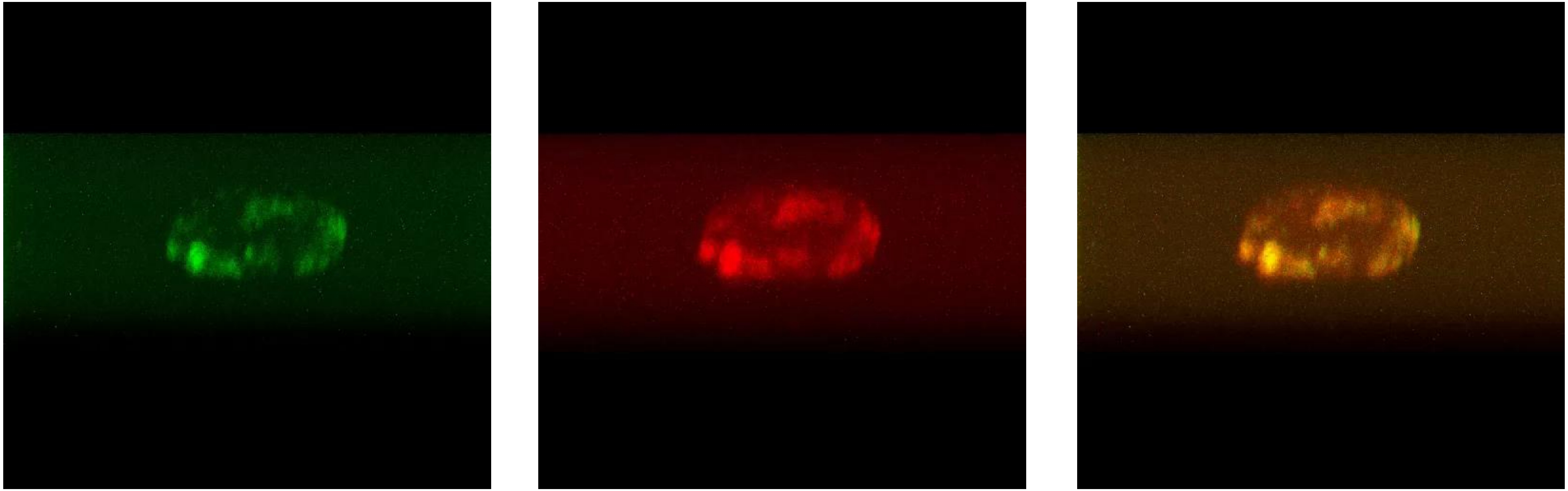

A B C

Single frames of 3D rendered confocal images (Imaris Bitplane) for DT40 B-lymphocyte cells stained for:

A) B-cell receptor, B) SPPLAT-deposited biotin, following incubation with HRP-tagged anti-(B-cell receptor) antibody, C) Merged images of A) and B). Cells were fixed, permeabilized and stained as described previously (19).

The cell is 10 micrometers in diameter.
